# Emigration patterns of motile cryptofauna and their implications for trophic functioning in coral reefs

**DOI:** 10.1002/ece3.9960

**Published:** 2023-03-28

**Authors:** Kennedy Wolfe, Amelia A. Desbiens, Peter J. Mumby

**Affiliations:** ^1^ Marine Spatial Ecology Lab, School of Biological Sciences and ARC Centre of Excellence for Coral Reef Studies University of Queensland Brisbane Queensland 4072 Australia

**Keywords:** biomass, emergent, movement, plankton, predation, RUBS, secondary production

## Abstract

Patterns of movement of marine species can reflect strategies of reproduction and dispersal, species' interactions, trophodynamics, and susceptibility to change, and thus critically inform how we manage populations and ecosystems. On coral reefs, the density and diversity of metazoan taxa are greatest in dead coral and rubble, which are suggested to fuel food webs from the bottom up. Yet, biomass and secondary productivity in rubble is predominantly available in some of the smallest individuals, limiting how accessible this energy is to higher trophic levels. We address the bioavailability of motile coral reef cryptofauna based on small‐scale patterns of emigration in rubble. We deployed modified RUbble Biodiversity Samplers (RUBS) and emergence traps in a shallow rubble patch at Heron Island, Great Barrier Reef, to detect community‐level differences in the directional influx of motile cryptofauna under five habitat accessibility regimes. The mean density (0.13–4.5 ind cm^−3^) and biomass (0.14–5.2 mg cm^−3^) of cryptofauna were high and varied depending on microhabitat accessibility. Emergent zooplankton represented a distinct community (dominated by the Appendicularia and Calanoida) with the lowest density and biomass, indicating constraints on nocturnal resource availability. Mean cryptofauna density and biomass were greatest when interstitial access within rubble was blocked, driven by the rapid proliferation of small harpacticoid copepods from the rubble surface, leading to trophic simplification. Individuals with high biomass (e.g., decapods, gobies, and echinoderms) were greatest when interstitial access within rubble was unrestricted. Treatments with a closed rubble surface did not differ from those completely open, suggesting that top‐down predation does not diminish rubble‐derived resources. Our results show that conspecific cues and species' interactions (e.g., competition and predation) within rubble are most critical in shaping ecological outcomes within the cryptobiome. These findings have implications for prey accessibility through trophic and community size structuring in rubble, which may become increasingly relevant as benthic reef complexity shifts in the Anthropocene.

## Graphical abstract

The density and diversity of species on coral reefs are greatest in dead coral and rubble, but this is driven by some of the smallest unseen individuals, limiting how accessible this energy is in the food web. Our study addresses this gap, showing how small‐scale patterns of movement influence prey accessibility, species' interactions, and community size structuring. Microhabitat‐scale differences can have appreciable impacts on communities and food webs, importantly as habitat degradation increases.

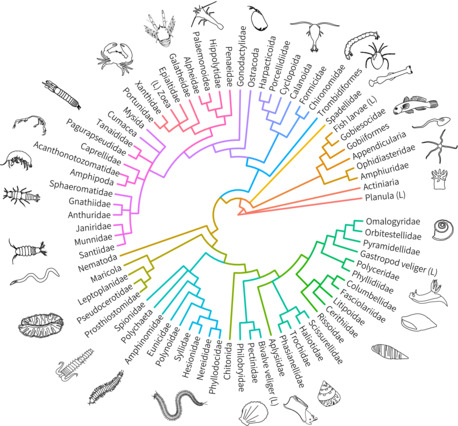

## 1 INTRODUCTION

The movement (or migration) of marine species is arguably best understood for large individuals that predictably migrate great distances to breeding or feeding grounds (Quinn & Brodeur, 1991). Yet, site fidelity and local population retention are common among marine fauna (Palumbi, 2004; Quinn & Brodeur, 1991), and for many species, the greatest distances are traveled during early life‐history stages as a lottery of larval dispersal in the vagaries of ocean currents (Allen et al., 2018). Indeed, one of the greatest known animal migrations on the planet is undertaken by some of the smallest individuals in the ocean—the vertical migration of zooplankton—which underpins marine functioning (Bandara et al., 2021; Hays, 2003). Understanding patterns of movement by marine species, regardless of their size, can provide valuable information on their reproductive strategies, distribution, species' interactions, food web dynamics, and susceptibility to disturbance (Allen et al., 2018; Cerca et al., 2018; Coker et al., 2012; Hays et al., 2016; Palmer et al., 1996), which critically inform how we manage species and ecosystems, including through marine park design (D'Aloia et al., 2017; Nagelkerken et al., 2015; Palumbi, 2004).

Adapted to extreme predation pressure, small marine invertebrates and fishes are typically characterized by high reproductive output and short generation times (Brandl et al., 2019; Coull, 1990). These biological traits can amount to high abundances, rapid population productivity, and local population retention, which fuels marine food webs and functioning from the bottom up (Brandl et al., 2018; Cerca et al., 2018; D'Aloia et al., 2015; Fraser et al., 2020). For example, despite their small adult size, cryptobenthic fishes can account for two‐thirds of the total larval pool of coral reef fishes, produce ~60% of consumed fish biomass, and contribute to ~90% of fish predation on coral reefs (Brandl et al., 2019; Mihalitsis et al., 2022). Local population retention coupled with rapid growth and extreme mortality underpins the fundamental role of cryptobenthic fishes in trophic productivity (Brandl et al., 2018, 2019). Yet, cryptobenthic fishes represent a comparatively small portion of the total diversity and density of coral reef cryptofauna, which is dominated by invertebrates (Stella et al., 2022; Wolfe, Kenyon, & Mumby, 2021).

On coral reefs, the density and diversity of motile cryptofauna increase from live coral to dead coral and rubble (Fraser et al., 2021a; Stella et al., 2022). As for cryptobenthic fishes, the productivity of motile invertebrate cryptofauna is high and can be up to 3 orders of magnitude greater in dead coral than in live coral (Fraser et al., 2021a; Kramer et al., 2014). Yet, the coral rubble interstices create a size‐limited habitat, providing high microhabitat complexity for small, lower trophic entities while leaving larger individuals exposed (Enochs & Manzello, 2012; Glynn & Enochs, 2011; Klumpp et al., 1988; Wolfe, Kenyon, & Mumby, 2021). The abundance of organisms typically decreases with increasing body size, with differences in this size spectra indicating variations in properties of ecosystem functioning such as predation and energy transfer (Currie, 1993; Damuth, 1981; Jennings & Mackinson, 2003; Jennings & Warr, 2003; Trebilco et al., 2013). High‐standing biomass and secondary production in rubble are therefore predominantly available in some of the smallest individuals, which limits how accessible this energy is to higher trophic levels (Fraser et al., 2021a; Heather et al., 2021; Stella et al., 2022). Predators able to forage successfully in rubble represent a critical stepping‐stone in reef trophodynamics, transferring energy from a highly productive but complex and somewhat inaccessible microhabitat (Kamen, 2020; Wolfe, Kenyon, & Mumby, 2021). There may indeed be a trophic mismatch between prey availability and the density of higher‐order predators within and around rubble beds (Kramer et al., 2016).

Food web models indicate that the increase in cryptofauna biomass in rubble may help to sustain coral reef functioning and fisheries productivity through enhanced resource availability under progressive changes in benthic habitat structure into the Anthropocene (Morais et al., 2020; Rogers et al., 2018). This poses an important question: what factors influence the bioavailability of rubble‐derived biomass? Coral rubble is indeed a natural but disturbed coral state, meaning the persistence of rubble communities depends on their ability to colonize this dynamic habitat type rapidly (Takada et al., 2016). Emigration rates of rubble communities to the novel substrate can be rapid, within days to weeks (Enochs et al., 2011; Takada et al., 2007; Valles et al., 2006; Wolfe & Mumby, 2020), driven by small‐scale patterns of movement within the benthos, or dispersal or recruitment from the water column (Callens et al., 2012; Cerca et al., 2018; Palmer, 1988). For emergent fauna, migration from the benthos at night results in a reshuffling of populations while predation risk is reduced (Alldredge & King, 1977; Takada et al., 2016), as biomass and productivity of nocturnal fishes is lower than diurnal species (Collins et al., 2022). Yet, for many rubble‐dwelling taxa, movement is likely to be highly constrained within the rubble cryptobenthos where they remain inconspicuous to top‐down predators (Takada et al., 2016), shaped by water depth (Takada et al., 2008, 2012), rubble morphology (Biondi et al., 2020), and the prevalence of sessile taxa, such as algae and sponges (Enochs, 2012; Gonzalez‐Gomez et al., 2018; Kramer et al., 2012; Logan et al., 2008; Tews et al., 2004).

Here, we address the bioavailability of coral reef cryptofauna in rubble based on small‐scale patterns of emigration. We adapted the accessibility of Rubble Biodiversity Samplers (RUBS), models used to standardize biodiversity sampling in rubble (Wolfe & Mumby, 2020), to explore the local movement patterns of rubble‐dwelling fauna, with inference to predation processes within and beyond the cryptobenthos. Five treatments were developed to detect community‐level differences in the directional influx of motile cryptofauna under various habitat accessibility regimes. We hypothesized that (1) cryptofauna abundance and biomass would increase when accessibility from the surface was blocked, as this would allow free interstitial movement and restrict top‐down predation; (2) cryptofauna abundance and biomass would decrease when all internal access was blocked, as fewer individuals would risk moving along the upper rubble surface and water column, and/or would be consumed in the process; (3) RUBS suspended above the benthos would host a subset of smaller, vagile individuals, dominated by taxa able to move freely beyond the benthos (Takada et al., 2016); and (4) emergent cryptofauna, collected using emergence plankton traps at night, would be similar to RUBS suspended above the substratum but distinct to those buried in the rubble benthos. Quantification of these small‐scale movement dynamics provides details on the interactions and functional processes driving enhanced cryptofauna density, diversity, and secondary productivity in rubble that may help delineate prey bioavailability in the Anthropocene.

## 2 MATERIALS AND METHODS

### 2.1 Study site and habitat accessibility treatments

**FIGURE 1:**
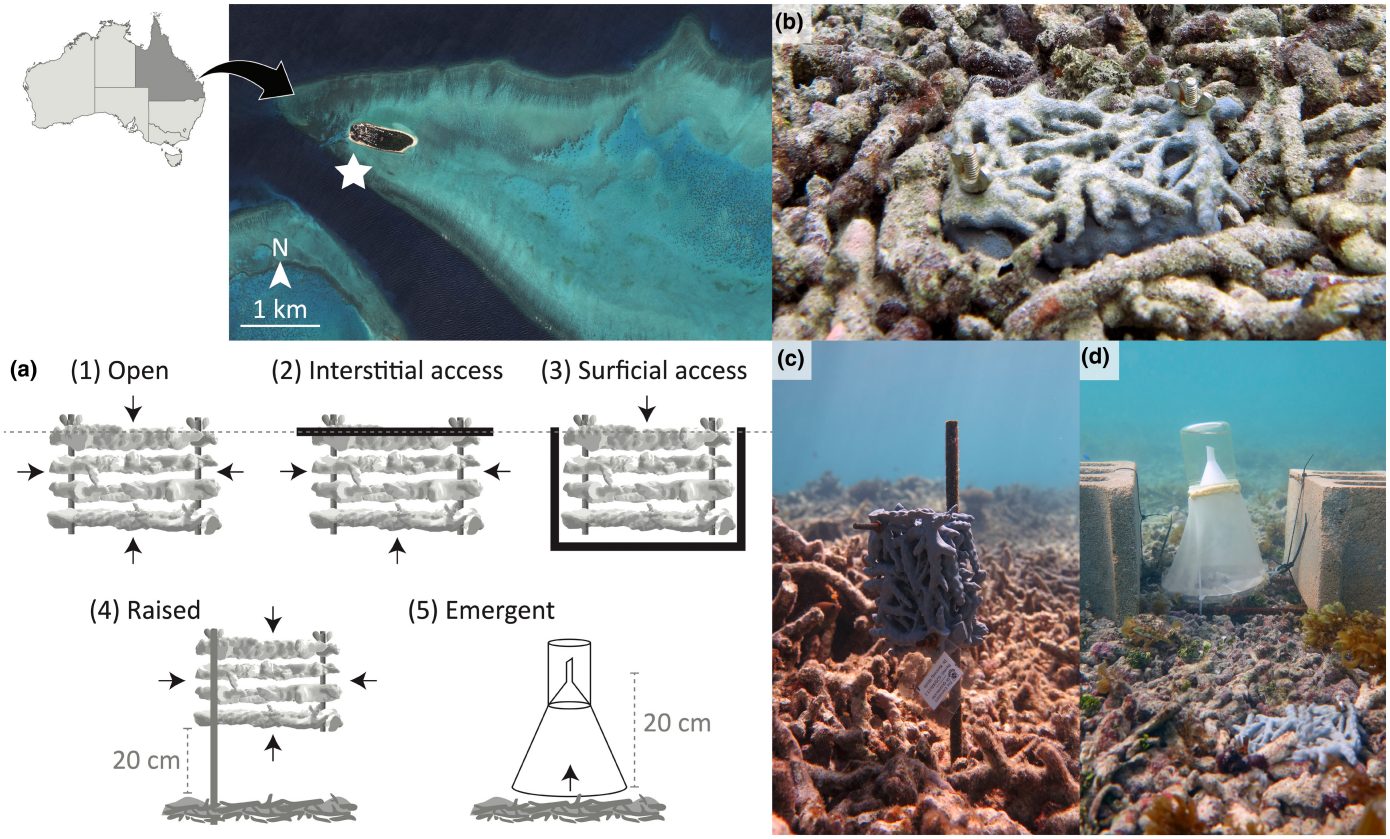
Location of the shallow rubble site at Heron Island (white star), and representation of (a) the five habitat accessibility treatments with pictures of (b) Rubble Biodiversity Samplers (RUBS) deployed in situ, (c) RUBS raised above rubble, and (d) RUBS alongside an emergence trap. For schematic: gray dotted line = rubble surface when RUBS are buried, and black arrows = directional accessibility in each treatment. Satellite image credit: IKONOS, NASA.

Fieldwork was conducted over several weeks (11th September to 5th October 2021) in a shallow (~3–5 m depth) reef slope site on the southern margin of Heron Island (−23°26.845′S, 151°54.732′E), Great Barrier Reef, Australia (Figure 1). This site is characterized by large rubble patches, comprised predominantly of branching rubble pieces, between stands of live *Acropora*. One rubble patch (~900 m^2^) was used in this experiment, accessed by boat with fieldwork conducted on SCUBA. Five treatments were established to quantify the local‐scale movement patterns of rubble‐dwelling fauna (Figure 1a). Four of these treatments were developed by modifying accessibility into Rubble Biodiversity Samplers (RUBS; https://www.thingiverse.com/thing:4176644/files; 140 × 90 × 80 mm), which are 3D‐printed models used to standardize biodiversity sampling of coral reef cryptofauna in rubble (Wolfe & Mumby, 2020). RUBS were modified to understand limitations on the directional influx and movement of cryptofauna within coral rubble patches using four treatments; (1) open (completely accessible), (2) interstitial access (top closed), (3) surficial access (sides and bottom closed), and (4) raised (above rubble substratum; Figure 1a–c). The fifth treatment involved a series of emergence plankton traps (Figure 1a,d), designed to target demersal cryptofauna that vertically migrate from within the rubble benthos at night, given emergent zooplankton biomass and diversity are greatest at night (Grutter et al., 2022; Sorokin & Sorokin, 2009, 2010). All collections were conducted under the Great Barrier Reef Marine Park Authority permit G20/44613.1.

### 2.2 RUBS deployment

RUBS with open, interstitial, and surficial access (*n* = 10 per treatment) were deployed haphazardly within the rubble patch in depressions in the rubble (~8 cm depth) made carefully by hand (Figure 1b). RUBS were never deployed near the patch edge nor in existing depressions. Only 4–6 RUBS were deployed at a time to allow sufficient time to process organisms live once collected (see below). The open treatment was unmodified with complete accessibility in and out of the model (Figure 1a). For the interstitial access treatment, the top surface of each RUBS was blocked with a thin transparent plastic cover so that organisms could not access the model from above, limiting movement to within the rubble interstices while eliminating top‐down predation potential (Figure 1a). For the surficial access treatment, the sides and bottom of each RUBS were blocked to limit within‐rubble movement, accessed only by the water column or exposed rubble surface while exposing the community to top‐down predation potential (Figure 1a). In each case, rubble from the immediate environment was used to infill gaps to ensure that RUBS surfaces were flush within the rubble patch (Figure 1b,d). All treatments were deployed randomly in the rubble bed with replicates staggered across the ~1‐month sampling period to reduce confounding spatial and temporal variation among treatments.

A fourth RUBS treatment was used to capture individuals that move freely beyond the rubble cryptobenthos. For this raised treatment, replicate RUBS (*n* = 8) were attached to stakes driven into the rubble bed. Models were raised ~20 cm off the substrate (Figure 1c), as small emergent organisms typically remain within 30 cm of the substrate (Alldredge & King, 1985; Kramer et al., 2013a). All RUBS were deployed with a small weight attached to the base and were preconditioned for ≥4 days in aquaria of flowing seawater, remaining submerged thereafter to maintain biofilm (Figure 1b), as done previously (Wolfe & Mumby, 2020).

### 2.3 RUBS retrieval and specimen processing

Each RUBS was retrieved after 7 days. RUBS were lifted from their depressions or freed from each stake, placed immediately into individual plastic collection bags underwater, and transferred in buckets to the laboratory where they were processed immediately (Wolfe & Mumby, 2020). To identify the cryptofauna community occupying each replicate, RUBS were dismantled and searched extensively for conspicuous fauna that could be easily removed using blunt probes, forceps, and plastic spoons and were placed into petri dishes for identification. Each RUBS layer was washed with pressurized freshwater over a tray to dislodge any fauna clinging to the surface. This was done several times with close inspection for motile fauna. Water from respective collection bags and wash trays was then poured through a 210 μm mesh sieve and all fauna retrieved for identification under a dissecting microscope. All organisms were identified to the lowest taxonomic resolution possible and measured to the nearest 0.25 mm in a scored dish. Taxonomic assistance was sought from relevant experts when unknown taxa were encountered (see Acknowledgements). Standard measurements were used; carapace width for crab‐like crustaceans or length for shrimp‐like crustaceans, shell length for mollusks, diameter for echinoderms with radial symmetry, and length for all types of worms. Sessile and encrusting biota were not quantified. All organisms were returned to their original site of collection postprocessing.

### 2.4 Emergent cryptofauna

Emergence plankton traps (Porter & Porter, 1977) were designed with an 18 cm ring base (surface area = 254.5 cm^2^) using 100 μm plankton mesh truncated to a positively buoyant plastic jar topped with an inverted funnel (Figure 1a,d). The vertical distance from the rubble substratum to the inside of the jar was ~20 cm, within the height range of small emergent taxa (Alldredge & King, 1985; Kramer et al., 2013a). All edges and attachments were sealed to minimize the risk of escapees and contamination from pelagic organisms.

Emergence traps were deployed over two consecutive nights (23–24th September 2021) within several days of the full moon to ensure that the effect of moonlight intensity on vertical migration was consistent (Alldredge & King, 1980). Replicate traps (*n* = 12) were placed directly onto flat rubble surface, weighted by adjoining stakes and cinder blocks (Figure 1d) before sunset (ca. 18:00 h), and were retrieved just after sunrise (ca. 07:00 h). For retrieval, traps were carefully released from their weights and returned to the surface while blocking the jar to avoid contamination of pelagic zooplankton. Samples were returned to the laboratory and processed immediately through a series of rinses into a 210 μm sieve, recorded, and returned to their site of collection, as above for RUBS samples.

### 2.5 Statistical analyses

A phylogenetic tree of all collected individuals was constructed at the lowest level of taxonomy possible using the open Tree of Life database (Maddison et al., 2007) and the *ggtree* function in R (Yu et al., 2017). To compare diversity among the five treatments, the Shannon–Weaver diversity index, inverse Simpson's diversity index, and Pielou's evenness coefficient were calculated using the *diversity* function of the *vegan* package in R (Oksanen et al., 2019). Differences in diversity indices among treatments were assessed using one‐way analysis of variance (ANOVA) in the base *stats* package of R (Chambers & Hastie, 1992), and the post‐hoc Tukey's HSD test using the *agricolae* package (de Mendiburu, 2021).

To compare data across treatments, density (ind cm^−3^) was calculated by standardizing total abundance data with the volume of RUBS (350 cm^3^). Data derived from emergence traps are typically presented in square meters (Alldredge & King, 1985; Kramer et al., 2013a), as they measure only at the rubble surface. For simplicity and comparability among treatments, we assumed a 1‐cm rubble depth to standardize the abundance of emergent fauna by emergence trap area to volume (254.5 cm^3^). Density conversions were performed at the lowest level of taxonomy possible within each replicate. Biomass was calculated per individual using existing length‐weight conversion factors for motile cryptofauna (Wolfe et al., 2020). Novel length‐weight relationships were established for *Linckia multifora* and the Gobiidae (Figure S1), which primarily comprised *Eviota* spp. for which no appropriate relationships were found on FishBase (www.fishbase.org). Where equations were not directly available, relationships of closely related taxa and/or those with similar morphologies were used as little data on the size and weight of coral reef cryptofauna are otherwise available. A minimum weight of 1 mg was set for larval forms and Actiniaria, as no reliable relationship was found (39 ind. total). Biomass data were standardized (mg cm^−3^) to sampler dimensions as for density and summed to represent a total wet standing biomass per replicate.

Community‐level differences in cryptofauna density and biomass were examined using multivariate Permutational Analysis of Variance (PERMANOVA) in PRIMER (v7) with 9999 permutations using Bray‐Curtis distribution matrices (Anderson et al., 2008). Treatment was used as a fixed factor and either density or biomass as the response variable at the lowest taxonomic resolution possible (namely to family). Significant differences were explored using pairwise and Similarity Percentage Analysis (SIMPER) tests. Density and biomass data were log‐transformed before analysis, and outcomes visualized using Principal Coordinates Analyses (PCoA) in PRIMER. All other statistics and visualizations were performed in R (R Core Team, 2019).

Size spectra of cryptofauna were determined for each treatment, as the linear relationship between log density and log size of all individuals within each sample. Linear relationships were developed using the base *stats* package of R (Chambers & Hastie, 1992) and resulting intercept and slope coefficients analyzed using PERMANOVA by treatment, as above, using Euclidean distances. For visualization, average size spectra metrics (slope, intercept, and *R* ^2^) within treatments were calculated.

## 3 RESULTS

### 3.1 Cryptofauna diversity

**FIGURE 2:**
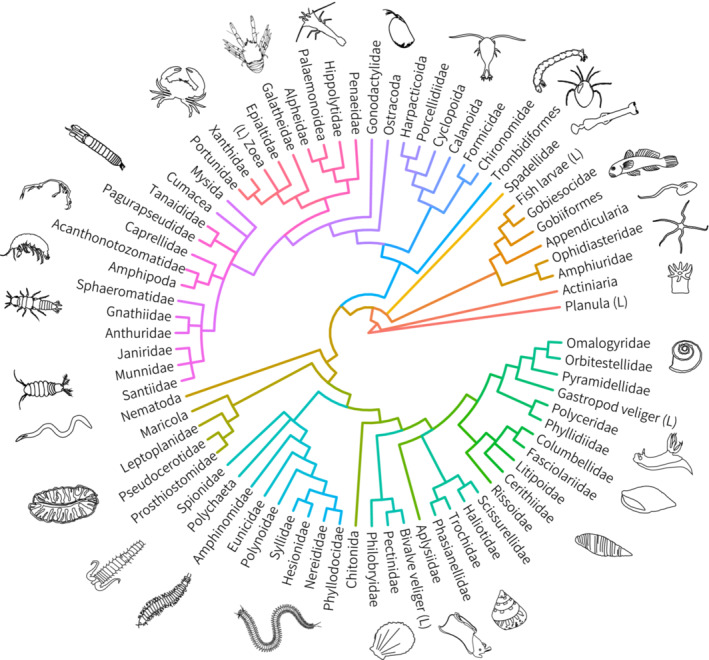
Phylogenetic tree of all rubble‐derived cryptofauna identified in the five habitat accessibility treatments. (L) indicates larval forms.

**FIGURE 3:**
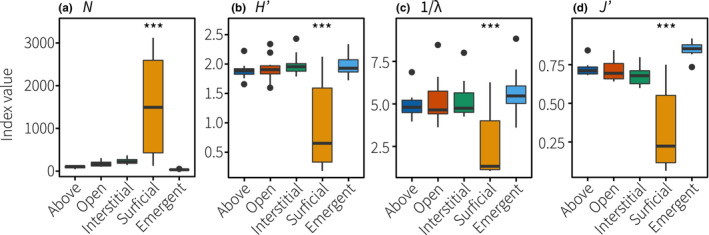
Average biodiversity indices for motile cryptofauna in the five habitat accessibility treatments, including (a) total abundance (*N*), (b) Shannon–Weaver diversity index (*H*′), (c) inverse Simpson's diversity index (1/*λ*), and (d) Pielou's evenness coefficient (*J*'). Asterisks denote significance (*p* < .001; Tukey's HSD tests).

We identified 20,849 individuals from 10 phyla, 19 classes, 46 orders, and 74 families of motile cryptofauna in coral rubble. Total diversity is expected to be much higher as not all individuals were identified to the family level, including a number of undescribed larval stages; planulae, decapod zoea, gastropod and bivalve veligers, and fish larvae (Figure 2). The Arthropoda dominated samples representing 91% of all individuals followed by the Mollusca at 8% of the total abundance. Of these, harpacticoid copepods, as well as Porcellididae (Copepoda) and Pyramidellidae (Gastropoda), were the most consistently abundant taxa. The total number of motile cryptofauna in samples was significantly greater in the surficial access treatment (Figure 3a), which hosted 75% of all individuals. This was skewed by an extremely high abundance of harpacticoids in the surficial access treatment (mean ± SD: 1405 ± 1194 ind.; range: 39–3009 ind.) compared with the remaining treatments (mean: 4.3–64 ind.). Variability was high for harpacticoids in the surficial access treatment with >1000 individuals found in six of the ten replicates (Figure S2). Yet, no clear community‐level or taxonomic differences were found in the remaining four samples with low harpacticoid abundance to explain this (i.e., no increased density or biomass of cryptic predators; Figure S2). The boom in harpacticoid density resulted in trophic simplification evident through lower species diversity indices and evenness coefficients in the surficial access treatment (Figure 3b–d). Diversity indices did not differ among the remaining four treatments, although species evenness was highest in communities collected using emergence traps (Figure 3d), which hosted just 2% of the total number of individuals counted.

### 3.2 Cryptofauna density

**FIGURE 4:**
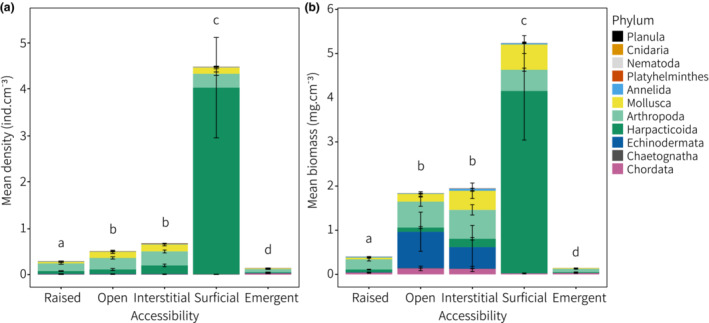
Mean (a) density (ind cm^−3^) and (b) biomass (mg cm^−3^) of rubble‐derived cryptofauna in the five habitat accessibility treatments. Data presented at the level of phylum with harpacticoid copepods separated from the Arthropoda. Letters that are the same do not differ (see pairwise results: Table S2).

**FIGURE 5:**
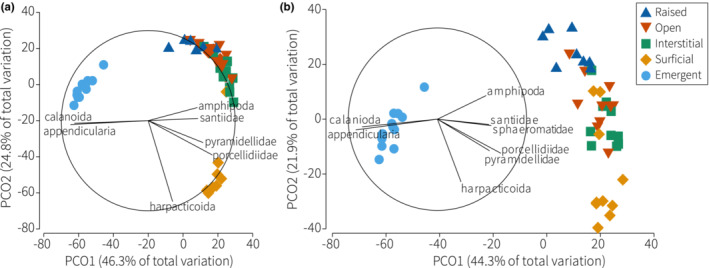
Principal Coordinate analysis (PCO) of the (a) density and (b) biomass of rubble‐derived cryptofauna in the five habitat accessibility treatments. Vectors shown only for top contributors (70% of similarities) within groups.

The mean density of cryptofauna ranged from 0.13 to 4.5 ind cm^−3^, which differed among treatments (PERMANOVA: *F*_4,49_ = 22.9, *p* < .001; Table S1). Mean cryptofauna density was 7‐ to 34‐fold greater in the surficial access treatment compared with the remaining treatments (pairwise: Table S2; Figure 4a). As above, this was heavily influenced by extremely high densities of harpacticoids in the surficial access treatment (mean ± SD; 4 ± 3.4 ind cm^−3^), which contributed to 62%–71% of dissimilarities among treatments (SIMPER: Table S3) and resulted in a distinct community dominated by one taxon (Figures 3, 4). Emergence traps captured a distinct community also, driven by comparatively high densities of Appendicularia and calanoid copepods (Figures 4a, 5a; Table S3). Appendicularia were not found in any other treatment. Emergence traps captured the lowest numbers of mollusks, and RUBS raised above the substratum had fewer mollusks than the three treatments buried in rubble (Tables S2, S3; Figure 4a). Specifically, lower densities of pyramidellid gastropods were found in the raised treatment, which explained up to 23% of the dissimilarities among treatments (Table S3). The mean density of amphipods was greatest in the raised treatment, explaining up to 15% of the dissimilarity among treatments (Table S3; Figure 5a). Densities of the Porcellidiidae were greatest in the interstitial and surficial access treatments (up to 21% of variation), while the Santiidae were greatest in the open and interstitial treatments (up to 18% of variation).

### 3.3 Cryptofauna biomass

The average wet weight of all individuals was 2 ± 18 mg (mean ± SD, range = 0.1–1710 mg) with a median of 1 mg per individual suggesting disproportionate numbers of tiny fauna. Mean weight per individual was greater in the open (4 ± 3.7 mg) and interstitial (3 ± 3.9 mg) access treatments compared with the raised (1 ± 3 mg), surficial (1 ± 7 mg), and emergence trap (1 ± 1 mg) treatments (Figure S3). Total biomass (mg cm^−3^) was dominated by the Arthropoda (68%) followed by the Echinodermata (14%), Mollusca (13%), and Chordata (4%). Mean biomass differed among all treatments, except between the open and interstitial access treatments (PERMANOVA: *F*_4,49_ = 15.5, *p* < .001; Tables S1, S2; Figures 4b, 5b), and was greatest in the surficial access treatment (5.2 ± 3.7 mg cm^−3^) and lowest in emergence traps (0.14 ± 0.03 mg cm^−3^).

Trends in biomass were similar to that of density (Figure 4a,b), weighted strongly by harpacticoid copepods, which contributed to near‐half (47%) of the total biomass across all samples and represented 79% of biomass in the surficial access treatment (Figure 5a,b). Mean harpacticoid biomass was 4.1 ± 3.5 mg cm^−3^ in the surficial access treatment, which was 22‐ to 277‐fold greater than the remaining treatments (0.015–0.19 mg cm^−3^) and explained up to 30% of the dissimilarities in biomass among treatments (SIMPER: Table S3; Figure 5b). The occasional presence of comparatively large sea stars (e.g., Ophidiasteridae: *Linckia multifora*), brittle stars (e.g., Amphiuridae), and cryptobenthic fishes (Gobiidae: *Eviota* spp. and *Callogobius* spp.) contributed to high biomass in the open and interstitial access treatments compared with the raised and emergence traps (Figure 4b) and explained up to 10% of variation among treatments (Table S3). Decapods (e.g., Galatheidae, Hippolytidae, Palaemonidae, Portunidae, and Xanthidae) contributed most to biomass in the open (0.27 ± 0.34 mg cm^−3^) and interstitial (0.28 ± 0.37 mg cm^−3^) access treatments and were not found in emergence traps. There was no notable increase in the density or biomass of larger predatory species (e.g., decapods and gobies) to explain the comparatively low abundance of harpacticoids in three replicates of the surficial access treatment (Figure S2).

The greatest gastropod biomass came from pyramidellid snails, which was highest in the interstitial access treatment (0.17 ± 0.12 mg cm^−3^) and explained over 11% of dissimilarities among treatments (Table S3). The Porcellidiidae and Amphipoda represented 21% and 17% of the mean biomass in the raised treatment, respectively, explaining up to 15% of variation in biomass among treatments (Table S3; Figure 5b). Rubble‐derived biomass was lowest in emergence traps (Figure 4b), influenced by the distinct community of Appendicularia and calanoid copepods (Figure 5b).

### 3.4 Cryptofauna size

**FIGURE 6:**
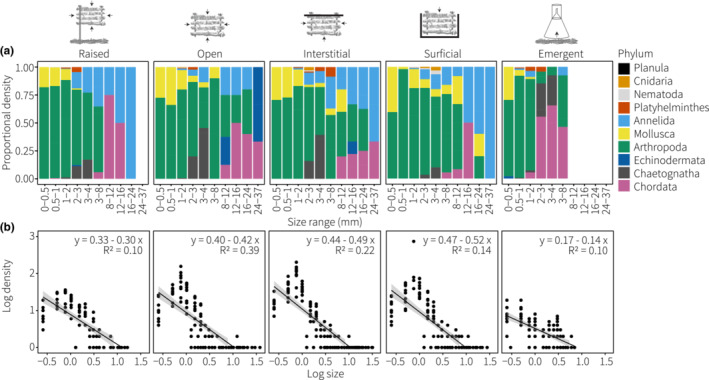
Size distributions of cryptofauna including (a) size classes per phylum, and (b) size spectra (log density~log size), among the five treatments. Slope, intercept, and *R* ^2^ provided on each plot.

The average size of all individuals was 0.88 mm (SD ± 1.02 mm, median = 0.75 mm, range = 0.25–37 mm). The Arthropoda dominated the smallest size classes while the Echinodermata and Chordata contributed most to large size classes (Figure 6a). Individuals >8 mm were never found in emergence traps while the largest individual in the raised RUBS treatment was a 16 mm long annelid worm from the Nereididae (Figure 6a). Few large chordates (e.g., Gobiidae) and arthropods (e.g., Decapoda) were found in the surficial access treatment compared with the open and interstitial treatments (Figure 6a).

Size spectra slopes, calculated from the relationships between cryptofauna density and body size, followed a typical pattern of greater densities of small individuals (Figure 6b). The best‐fit relationship between cryptofauna size and density was found in the open access treatment (*R* ^2^ = .39), though linear relationships were generally weak (*R* ^2^ = .10–.22) owing to a peak in the density of individuals ~0.75–1 mm compared with the smallest size classes <0.5 mm (Figure 6b). Size spectra of cryptofauna differed among the five treatments (PERMANOVA: *F*_4,49_ = 14.6, *p* < .001; Table S1), with all RUBS treatments exhibiting a steeper and longer slope compared with emergence traps (Figure 6b). The raised treatment further differed from the interstitial and surficial treatments, which had the steepest size spectra slopes (pairwise: Table S2, Figure 6b).

## 4 DISCUSSION

Coral reef biodiversity and secondary productivity can be greatest in dead coral and rubble (Fraser et al., 2021a; Kramer et al., 2014), driven by high abundances and population turnover of some of the smallest metazoan reef fauna (Wolfe, Kenyon, & Mumby, 2021). Understanding patterns in the bioavailability of motile cryptofauna in rubble–an eroded reef condition–is critical to predicting outcomes of reef degradation as we move deeper into the Anthropocene (Masucci et al., 2021). We documented distinct community‐level differences in the movement patterns of motile cryptofauna under five habitat accessibility regimes in rubble. Specifically, we found a 7‐ to 34‐fold increase in cryptofauna density when all interstitial access within rubble was blocked, driven by the rapid immigration and proliferation of one taxon (harpacticoid copepods) from the rubble surface. Decapods, cryptobenthic fishes, and echinoderms contributed to high relative biomass in the open and interstitial access treatments, indicating larger cryptic taxa are limited within the rubble interstices with size‐based constraints on movement at or beyond the rubble surface. Emergent faunae were a distinct community but had the lowest density and biomass, which suggests limitations on secondary production from rubble at night. These data help to understand the small‐scale patterns of emigration, species' interactions, and bioavailability of motile cryptofauna in rubble, and highlight how slight alterations to microhabitat structure can rapidly alter ecological outcomes in coral reefs.

### 4.1 Proliferation of harpacticoid copepods

Our primary hypothesis that the surficial access treatment would host a lower density and biomass of cryptofauna owing to restricted interstitial movement and increased predation risk from higher‐order taxa beyond the benthos was not supported. We observed an extreme influx of thousands of harpacticoid copepods when RUBS could only be accessed from the surface. This response explained most of the differences in cryptofauna density among treatments. Harpacticoids are ubiquitous in benthic marine habitats (Buffan‐Dubau & Carman, 2000; Coull, 1999; Klumpp et al., 1988; Kramer et al., 2017), abundant in rubble (Callens et al., 2012; Fraser et al., 2021a; Kramer et al., 2014), and are a common, high‐quality food item for coral reef invertivores that can avail of small prey (Kamen, 2020; Kramer et al., 2013b). Harpacticoids were indeed among the most abundant taxa in all treatments, as expected (Wolfe & Mumby, 2020), so it is intriguing that the surficial access treatment triggered such an exceptional degree of colonization. We postulate that this was due to a combination of conspecific cues, a reduction in interstitial predation, and/or physical or biogeochemical conditions that created the treatment itself.

Harpacticoids typically have short life spans, and rapid rates of reproduction and development of several weeks (Dahms & Qian, 2004; Green et al., 1995). Given no nauplii were found and the mean size of harpacticoids was 0.75 mm (SD ± 0.06 mm; *n* = 15,225), it is most probable that the influx of harpacticoids to RUBS within 7 days of deployment resulted from surface‐derived immigration not rapid reproduction, recruitment, and postsettlement growth. Harpacticoids are indeed vagile and can rapidly colonize newly available substrates from the water column and interstitially (Atilla et al., 2003; Callens et al., 2012; Chertoprud et al., 2005; Walters & Bell, 1994). Neither direction‐based limitations in harpacticoid movement nor ontogeny seems to explain their proliferation in the surficial access treatment.

The mean biomass of harpacticoids in the surficial access treatment was 22‐ to 277‐fold greater than that found in the remaining treatments, and at least two orders of magnitude greater than estimates of harpacticoids in other benthic habitats (Kramer et al., 2012, 2014), and of pelagic zooplankton (Roman et al., 1990), on the Great Barrier Reef. It seems our data present a method to drastically and rapidly enhance harpacticoid productivity in situ, which would have implications for reef trophodynamics. Rubble‐based harpacticoids represent critical lower trophic components of coral reef food webs (Kamen, 2020; Kramer et al., 2014), but converse to expectation, harpacticoids did not benefit when exposure to top‐down predators was limited (i.e., RUBS surface‐blocked). Estimates of copepod consumption by reef fishes are extremely high (Kramer et al., 2013b), but rates of predation at this trophic level (Kamen, 2020) seem insufficient to diminish the short generation times, rapid colonization rates, and high population productivity of small marine invertebrates (Coull, 1990). In fact, these traits impart high resilience of the cryptofauna and meiofauna to habitat disturbance and climate change (Cerca et al., 2018; Schratzberger & Ingels, 2018; Schratzberger & Somerfield, 2020; Timmers et al., 2021; Wolfe, Deaker, et al., 2021; Zeppilli et al., 2015), which infers their ecological roles may amplify in a future ocean.

While exposure to higher‐order predators did not alter the cryptofauna community, the extreme influx of harpacticoids when interstitial access was blocked suggests they experience very high rates of predation from within rubble naturally, fuelled by high population productivity and community turnover. Indeed, copepods exhibit predator avoidance in their benthic (Dethier, 1980; Itoh & Nishida, 2013) and pelagic (Alldredge & King, 1977) interactions. Small‐bodied cryptofauna (specifically harpacticoids) may have risked leaving the rubble interstices to colonize newly available space from the rubble surface as larger predatory cryptobenthic fishes and decapods were comparatively low in this treatment. Within‐rubble competition and predation may have been alleviated allowing small individuals the chance to proliferate. Competition and predation within rubble seem critical in shaping lower trophic level outcomes, as found for coral‐associated taxa (Stier & Leray, 2014). Why this would exclusively benefit harpacticoids is unclear unless they are the preferred food of cryptic predators, which may be the case for some cryptobenthic fishes (Brandl et al., 2018, 2020). If predation was the primary driver of this outcome for harpacticoids in the surficial access treatment, one would expect the few replicates with lower harpacticoid densities to have a distinct predator presence or community structure, but this was not the case (see Figure S2). The influx of harpacticoids does not seem to be solely predator‐driven, but explicit details on rubble‐based food webs are lacking to inform this.

We cannot distinguish from our data whether the disproportionate density of harpacticoids in the surficial access treatment was due to rapid immigration on the day of sampling, or a gradual aggregative accumulation over the week‐long deployment facilitated by the lack of predators and/or a cumulative increase in conspecific cues in this treatment. It seems most plausible that a lower predator density made it possible to aggregate at higher densities with higher sociochemical cue concentrations that expedited rates of influx. Species‐specific cues and interactions influence spatial patterns in harpacticoids (Chandler & Fleeger, 1987), a taxon that respond rapidly to pheromones and changes in their chemical environment (Kelly et al., 1998; Sibly et al., 2000; Walker, 1979). It is possible that intra‐ (e.g., pheromones) and/or interspecific (e.g., no predator scent) cues triggered harpacticoid aggregations in our largely encased surficial access treatments.

In addition, a low‐flow environment with altered biogeochemistry (e.g., oxygen content, pH) was likely generated when the RUBS sides and bottoms were blocked, creating an environmental cue that stimulated harpacticoid movement across the rubble surface. Copepods dominate benthic communities under low‐flow conditions in structurally complex microhabitats (Palmer, 1988), with variable colonization rates based on taxonomy and habitat associations (Callens et al., 2012). This presents an interesting outcome regarding chemical cues, water flow, and biogeochemical processes in rubble, which may shape and possibly even fuel ecological and trophodynamic outcomes. However, it is important to note that the results for harpacticoids here provide evidence of trophic simplification following minor alterations to microhabitat structure or accessibility, a concern for future reef functioning (Alvarez‐Filip et al., 2015; Kroeker et al., 2011; Ullah et al., 2018). It therefore seems imperative to characterize flow and biogeochemical parameters within‐rubble patches of varying typologies.

### 4.2 Nighttime bioavailability of cryptofauna

Emergent zooplankton is a distinct group of marine invertebrates that migrate in response to external factors including moon phase, tides, water flow, and temperature (Alldredge & King, 1980, 1985; Cortes et al., 2017; Esquivel‐Garrote & Morales‐Ramírez, 2020; Kramer et al., 2013a; Porter & Porter, 1977). While our emergence traps captured the lowest density (0.13 ± 0.03 ind cm^−3^) and biomass (0.15 ± 0.03 mg cm^−3^) of cryptofauna from rubble (mean ± SD; *n* = 12), these values are within the ranges of those reported previously for emergent zooplankton from other benthic habitat types on the Great Barrier Reef (Alldredge & King, 1977; Sorokin & Sorokin, 2009, 2010). Indeed, the emergent community here was distinct from that of the remaining treatments using RUBS, including those raised above the benthos. This reinforces that emergent zooplankton has distinct ecological and trophodynamic roles (Gorsky & Fenaux, 1998; Grutter et al., 2022; Kramer et al., 2013a) with links to specific higher‐order taxa (Sorokin & Sorokin, 2009, 2010), predominantly cardinalfishes (Apogonidae) at night (Collins et al., 2022). Though substantially reduced biomass and biomass production of nocturnal compared with diurnal reef fishes may indeed be attributed to resource limitation (Collins et al., 2022).

Community‐level differences in the emergence trap treatment were driven by higher densities of the Appendicularia and Calanoida. Calanoid copepods are an abundant group of emergent zooplankton (Alldredge & King, 1977), indeed were 6‐ to 15‐fold more abundant in the emergence traps compared with the remaining treatments, and contributed most to the total biomass of emergent fauna. The mean density of Appendicularia (0.02 ± 0.01 ind cm^−3^) in emergence traps was greater than in previous reports on the Great Barrier Reef (Alldredge & King, 1977) and elsewhere (Esquivel‐Garrote & Morales‐Ramírez, 2020). Appendicularia are predominantly pelagic (Gorsky & Fenaux, 1998) but have been found in demersal plankton samples on the Great Barrier Reef (e.g., Grutter et al., 2022). The absence of Appendicularia from any RUBS treatment, including those suspended above the benthos, suggests benthic larvaceans are not common near the rubble surface and may reside deeper in the rubble interstices during the day, at least beyond the RUBS depth profile (~10 cm). However, little information is available on benthic larvaceans to explore this hypothesis.

The zooplankton commonly comprises the early life‐history stages of many benthic taxa that disperse through the water column (Esquivel‐Garrote & Morales‐Ramírez, 2020; Palmer, 1988; Warwick et al., 1986). We found crustacean zoea, gastropod, and bivalve veligers, and fish larvae in emergence traps, which highlights the importance of rubble in supporting cryptofauna ontogenesis (Wolfe, Kenyon, & Mumby, 2021). However, little is known of the full life histories of the majority of invertebrate cryptofauna (for cryptobenthic fishes see: Brandl et al., 2019), including patterns of larval retention and settlement. Targeted surveys and experiments are required to understand cryptofauna reproduction, dispersal, and marine connectivity, which would benefit from genome scanning approaches (Cerca et al., 2018).

### 4.3 Trophic structure and functioning in rubble biomes

It has long been recognized that invertebrate biomass and energy transfers exceed that of reef fish communities (Glynn & Enochs, 2011; Opitz, 1993). Biomass data were extrapolated to a range of 0.01 t ha^−1^ for fauna found in emergence traps to 1.04 t ha^−1^ in RUBS with restricted interstitial accessibility. After just 7 days of RUBS deployment, standing rubble‐derived biomass was within the ranges of reef fish biomass (0.45–2.22 t ha^−1^; Collins et al., 2022), and exceeded previous measures of cryptofauna (Kramer et al., 2014) and zooplankton (Sorokin & Sorokin, 2009, 2010) production on the Great Barrier Reef. Notably, these results are likely to represent a portion of natural rubble communities. Longer RUBS deployments would be expected to capture a greater density and biomass of cryptofauna by at least two‐fold (as in: Wolfe & Mumby, 2020), and our mesh size of 210 μm would have excluded a considerable density of even smaller individuals by at least an order of magnitude (as in: Kramer et al., 2012), which would cumulatively contribute to higher biomass and productivity (Kramer et al., 2013b). Yet, even within a short experimental time frame, our data underscore the propensity of cryptofauna to rapidly colonize newly available dead coral space (Callens et al., 2012), a trait that underpins coral reef food webs (Fraser et al., 2021a; Kramer et al., 2014), and is predicted to help sustain reef food webs as reef complexity deteriorates (Morais et al., 2020; Rogers et al., 2018; Wolfe, Kenyon, & Mumby, 2021).

The range in density and biomass data across the five habitat accessibility treatments emphasizes the complexities in attempting to quantify coral reef trophodynamics involving rubble‐derived biomass. Our study adds evidence to the high biomass and secondary production of motile cryptofauna in rubble (as in: Fraser et al., 2021a), at least in shallow (~3–5 m depth) rubble patches typical of the reef slope. We refrained from calculating secondary production owing to the limitations of using ash‐free dry‐weight conversions for diverse invertebrate communities that span a breadth of morphologies (Edgar, 1990), and the requirement to sacrifice animals. Previous estimates suggest that secondary production of coral reef cryptofauna is primarily influenced by microhabitat type (Fraser et al., 2020), with a 1–3 order of magnitude increase in motile invertebrate productivity predicted as live coral is replaced by turf‐covered dead coral and rubble (Fraser et al., 2021a; Kramer et al., 2014). We add greater complexity to these outcomes by demonstrating how small‐scale alterations to microhabitat accessibility can have appreciable influences on the density, biomass, and likely, trophic ecology of coral reef cryptofauna in rubble.

Per individual biomass was greatest in treatments with open and interstitial access (see Figure S3), skewed by low numbers of larger‐bodied echinoderms (e.g., *Linckia multifora*, Amphiuridae), decapods (e.g., Galatheidae, Hippolytidae, Palaemonidae, Portunidae, and Xanthidae), and fishes (Gobiesocidae and Gobiidae; e.g., *Eviota* and *Callogobius*), as common of rubble biomes (Stella et al., 2022; Wolfe et al., 2020; Wolfe, Kenyon, & Mumby, 2021). Interestingly, the mean density and biomass of cryptofauna did not differ between open and interstitial access (i.e., surface‐blocked) treatments, confirming that exposure to top‐down predation is not a limiting factor of the rubble‐dwelling cryptofauna (Fraser et al., 2020; Stella et al., 2022), despite being heavily preyed upon (Kamen, 2020). Invertivorous fishes frequently inspect RUBS when deployed (Wolfe, pers. obs.), and species of the Apogonidae, Balistidae, Gobiidae, Labridae, Muraenidae, Plesiopidae, Scorpaenidae, Serranidae, and Syngnathidae have been found in RUBS collections previously (Wolfe & Mumby, 2020), but their influence on cryptofauna was not detected here.

While external higher‐order predators may have little impact on rubble‐derived productivity (Fraser et al., 2020; Stella et al., 2022), there are clearly size limitations to life in rubble. Our data indicate that those required to move along or above the benthos to colonize sampling units were dominated by small‐bodied taxa with the capacity to swim and disperse, as found previously (Takada et al., 2016). The lack of larger cryptofauna in these treatments suggests they either avoid moving along the rubble surface or are readily consumed in the process. Larger taxa may prefer to inhabit rubble via the passive benthic pathway as they have limited mobility (i.e., nonswimming) and/or exhibit high degrees of predator avoidance behavior (Takada et al., 2016). Decapods and cryptobenthic fishes are indeed fundamental prey of coral reef fishes (Brandl et al., 2018, 2019; Glynn & Enochs, 2011; Kramer et al., 2015; Leray et al., 2012) and must remain cryptic among the reef and rubble infrastructure to avoid mortality. It seems of interest to test how rubble piece size and morphology, patch depth, and consequently, varying degrees of interstitial space, influence emigration patterns and size structuring of the cryptofauna in rubble biomes, and how they may determine the transfer of energy to higher‐order taxa in a current and future ocean.

Many larger members of the cryptofauna are predatory (Mihalitsis et al., 2022; Wolfe, Kenyon, & Mumby, 2021), and the exceptional influx of harpacticoids in their absence indicates that predators within the rubble interstices are likely to have a stronger impact on trophic outcomes at this level of reef functioning than those beyond the cryptobenthos. Indeed, the best‐fit and steepest size spectra slopes were found in interstitially‐open treatments. This demonstrates longer food chains supported by small predator–prey ratios (Fraser et al., 2021b; Heather et al., 2021; Jennings & Mackinson, 2003; Jennings & Warr, 2003), and thus, the importance of predation processes within rubble. However, ecological interactions within‐rubble communities are poorly characterized and empirical data on diets and food webs (e.g., stable isotope analysis, gut contents, DNA metabarcoding) are required. Our data highlight the importance of considering and characterizing the direct role of cryptic predators (e.g., decapods, polychaetes, and mollusks) when attempting to quantify trophic outcomes in coral reefs (Glynn & Enochs, 2011; Mihalitsis et al., 2022). Rubble‐dwelling predators would, after all, be expected to have the greatest accessibility to rubble‐derived biomass. Yet, how available this lower trophic biomass is to higher‐order fishes requires close attention as disproportionate densities of small fauna in rubble may shift trophic functioning as coral reefs change in the Anthropocene (Wolfe, Kenyon, & Mumby, 2021).

## AUTHOR CONTRIBUTIONS

**Kennedy Wolfe:** Conceptualization (equal); data curation (lead); formal analysis (lead); funding acquisition (equal); investigation (lead); methodology (lead); project administration (lead); resources (equal); software (equal); validation (lead); visualization (lead); writing – original draft (lead); writing – review and editing (equal). **Amelia A. Desbiens:** Data curation (supporting); formal analysis (supporting); investigation (supporting); methodology (supporting); validation (supporting); writing – review and editing (equal). **Peter J. Mumby:** Conceptualization (equal); funding acquisition (equal); methodology (supporting); resources (equal); software (equal); supervision (lead); writing – review and editing (equal).

## CONFLICT OF INTEREST STATEMENT

The authors declare no conflicts of interest.

### OPEN RESEARCH BADGES

This article has earned Open Data and Open Materials badges. Data are available in the Dryad digital repository https://doi.org/10.5061/dryad.0k6djhb4k (open data). RUBS models are available on the Thingyverse platform https://www.thingiverse.com/thing:4176644/files (open materials).

## Supporting information


Appendix S1.

## Data Availability

All data pertaining to this study are archived and available in the Dryad digital repository https://doi.org/10.5061/dryad.0k6djhb4k (Wolfe et al., 2023). No novel code was used.
