# Supplementary material for: Emigration patterns of motile cryptofauna and their implications for trophic functioning in coral reefs
Source: Ecol Evol. 2023 Mar 28;13(3):e9960. doi: 10.1002/ece3.9960 (PMC10049886; doi:10.1002/ece3.9960)
Supplement: Supplementary file 1 — Appendix S1. [file ECE3-13-e9960-s001.docx]

**Figure S1:** Length-weight relationships of (A) *Linckia multifora* (data derived from n = 14 individuals collected from Heron Island, Great Barrier Reef, Australia, in October 2022) and (B) *Eviota* sp. (Gobiidae; data from n = 114 individuals collected in Koror, Palau, Western Micronesia, available in Wolfe et al. 2020 and Stella et al. 2022).

Stella, J. S., K. Wolfe, G. Roff, A. Rogers, M. Priest, Y. Golbuu, and P. J. Mumby. 2022. Functional and phylogenetic responses of motile cryptofauna to habitat degradation. Journal of Animal Ecology

Wolfe, K., A. Desbiens, J. Stella, and P. J. Mumby. 2020. Length–weight relationships to quantify biomass for motile coral reef cryptofauna. Coral Reefs **39**:1649-1660.

**Figure S2:** Outcomes in the surficial access treatment were subsampled to explore community drivers of variable harpacticoid density in this treatment, including cryptofauna (A) density per replicate showing low density of harpacticoids (green bars) in replicates 1, 7 and 10, (B) biomass excluding harpacticoids, which did not vary in these replicates (black arrows). PCO analyses of (C) density and (D) biomass data excluding harpacticoids did not show clear community differences in replicates 1, 7 or 10 (red points).

**Figure S3:** Mean biomass per individual (mg.ind^-1^.cm^-3^) in the five accessibility treatments.

**Table S1:** Summary results of PERMANOVA tests on the (A) density and (B) biomass of cryptofauna by the five collection method treatments.

|  | Source | df | SS | MS | Pseudo-F | P(perm) |
| --- | --- | --- | --- | --- | --- | --- |
| (A) Density | Treatment | 4 | 78145 | 19536 | 22.858 | **<0.001** |
|  | Res | 45 | 38461 | 854.69 |  |  |
|  | Total | 49 | 116610 |  |  |  |
| (B) Biomass | Treatment | 4 | 70587 | 17647 | 15.509 | **<0.001** |
|  | Res | 45 | 51202 | 1137.8 |  |  |
|  | Total | 49 | 121790 |  |  |  |
| (C) Size spectra | Treatment | 4 | 1.7293 | 0.43232 | 14.578 | **<0.001** |
|  | Res | 45 | 1.3345 | 0.02966 |  |  |
|  | Total | 49 | 3.0638 |  |  |  |

**Table S2:** Pairwise PERMANOVA results on the effect of sampling treatment on rubble cryptofauna (A) density and (B) biomass. Significant values in bold.

|  | **Groups** | **t** | ***p* value** | **Unique perms** |
| --- | --- | --- | --- | --- |
| (A) Density | open, raised | 2.677 | **<0.001** | 8890 |
|  | open, interstitial | 1.268 | 0.151 | 9466 |
|  | open, surficial | 3.401 | **<0.001** | 9385 |
|  | open, emergent | 6.687 | **<0.001** | 9852 |
|  | raised, interstitial | 3.334 | **<0.001** | 8947 |
|  | raised, surficial | 3.787 | **<0.001** | 8969 |
|  | raised, emergent | 6.108 | **<0.001** | 9576 |
|  | interstitial, surficial | 3.157 | **0.002** | 9419 |
|  | interstitial, emergent | 7.452 | **<0.001** | 9842 |
|  | surficial, emergent | 6.043 | **<0.001** | 9851 |
| (B) Biomass | raised, open | 2.288 | **<0.001** | 8865 |
|  | raised, interstitial | 2.587 | **<0.001** | 8855 |
|  | raised, surficial | 2.983 | **<0.001** | 8846 |
|  | raised, emergent | 5.621 | **<0.001** | 9527 |
|  | open, interstitial | 0.996 | 0.478 | 9354 |
|  | open, surficial | 2.181 | **0.001** | 9429 |
|  | open, emergent | 5.541 | **<0.001** | 9841 |
|  | interstitial, surficial | 1.937 | **0.001** | 9424 |
|  | interstitial, emergent | 6.096 | **<0.001** | 9833 |
|  | surficial, emergent | 6.098 | **<0.001** | 9837 |
| (C) Size spectra | raised, open | 1.605 | 0.111 | 8879 |
|  | raised, interstitial | 2.690 | **0.012** | 8929 |
|  | raised, surficial | 2.442 | **0.022** | 8883 |
|  | raised, emergent | 3.053 | **0.001** | 9572 |
|  | open, interstitial | 1.324 | 0.186 | 9435 |
|  | open, surficial | 1.446 | 0.153 | 9410 |
|  | open, emergent | 6.294 | **<0.001** | 9856 |
|  | interstitial, surficial | 0.559 | 0.622 | 9405 |
|  | interstitial, emergent | 8.279 | **<0.001** | 9868 |
|  | surficial, emergent | 6.225 | **<0.001** | 9855 |

**Table S3:** Results of SIMPER analyses on the effect of sampling treatment on the density and biomass rubble cryptofauna. SIMPER analyses performed using Bray-Curtis matrices with average similarity, dissimilarity, and contribution stated (top 70%). Note, values for biomass are low but never true zero.

**Density**

**Raised**: average similarity = 62.97

| Family | Av.Abund | Av.Sim | Sim/SD | Contrib% | Cum.% |
| --- | --- | --- | --- | --- | --- |
| Porcellidiidae | 0.08 | 19.88 | 2.53 | 31.58 | 31.58 |
| Amphipoda | 0.06 | 16.61 | 2.60 | 26.38 | 57.95 |
| Harpacticoida | 0.06 | 15.98 | 4.51 | 25.38 | 83.34 |

**Open**: average similarity = 60.97

| Family | Av.Abund | Av.Sim | Sim/SD | Contrib% | Cum.% |
| --- | --- | --- | --- | --- | --- |
| Porcellidiidae | 0.10 | 15.73 | 3.73 | 25.80 | 25.80 |
| Pyramidellidae | 0.11 | 12.37 | 1.83 | 20.29 | 46.10 |
| Santiidae | 0.07 | 11.26 | 2.29 | 18.48 | 64.57 |
| Harpacticoida | 0.09 | 11.14 | 1.49 | 18.28 | 82.85 |

**Interstitial**: average similarity = 66.91

| Family | Av.Abund | Av.Sim | Sim/SD | Contrib% | Cum.% |
| --- | --- | --- | --- | --- | --- |
| Harpacticoida | 0.17 | 19.22 | 2.39 | 28.73 | 28.73 |
| Porcellidiidae | 0.12 | 14.68 | 4.95 | 21.93 | 50.66 |
| Pyramidellidae | 0.13 | 13.53 | 2.65 | 20.21 | 70.87 |

**Surficial**: average similarity = 53.64

| Family | Av.Abund | Av.Sim | Sim/SD | Contrib% | Cum.% |
| --- | --- | --- | --- | --- | --- |
| Harpacticoida | 1.31 | 37.53 | 1.25 | 69.97 | 69.97 |
| Porcellidiidae | 0.15 | 6.14 | 1.66 | 11.44 | 81.41 |

**Emergent**: average similarity = 63.10

| Family | Av.Abund | Av.Sim | Sim/SD | Contrib% | Cum.% |
| --- | --- | --- | --- | --- | --- |
| Calanoida | 0.04 | 23.63 | 4.29 | 37.44 | 37.44 |
| Appendicularia | 0.02 | 13.27 | 2.58 | 21.03 | 58.48 |
| Harpacticoida | 0.01 | 6.87 | 1.33 | 10.88 | 69.36 |
| Spadellidae | 0.01 | 4.70 | 1.55 | 7.44 | 76.80 |

**Raised vs. Open**: average dissimilarity = 50.09

| Family | Av.abund 1 | Av.abund 2 | Av.diss | Diss/sd | Contrib% | Cum.% |
| --- | --- | --- | --- | --- | --- | --- |
| Pyramidellidae | 0.03 | 0.11 | 11.40 | 1.37 | 22.77 | 22.77 |
| Santiidae | 0.00 | 0.07 | 9.18 | 2.46 | 18.33 | 41.10 |
| Harpacticoida | 0.06 | 0.09 | 7.33 | 1.17 | 14.63 | 55.73 |
| Porcellidiidae | 0.08 | 0.10 | 6.09 | 1.31 | 12.17 | 67.89 |
| Amphipoda | 0.06 | 0.03 | 4.32 | 1.60 | 8.62 | 76.51 |

**Raised vs. Interstitial**: average dissimilarity = 51.75

| Family | Av.abund 1 | Av.abund 2 | Av.diss | Diss/sd | Contrib% | Cum.% |
| --- | --- | --- | --- | --- | --- | --- |
| Harpacticoida | 0.06 | 0.17 | 12.42 | 1.93 | 24.01 | 24.01 |
| Pyramidellidae | 0.03 | 0.13 | 11.42 | 1.57 | 22.06 | 46.07 |
| Santiidae | 0.00 | 0.07 | 7.46 | 1.56 | 14.41 | 60.48 |
| Porcellidiidae | 0.08 | 0.12 | 6.49 | 1.36 | 12.53 | 73.02 |

**Open vs. Interstitial**: average dissimilarity = 37.00

| Family | Av.abund 1 | Av.abund 2 | Av.diss | Diss/sd | Contrib% | Cum.% |
| --- | --- | --- | --- | --- | --- | --- |
| Harpacticoida | 0.09 | 0.17 | 9.17 | 1.52 | 24.79 | 24.79 |
| Pyramidellidae | 0.11 | 0.13 | 7.89 | 1.29 | 21.33 | 46.12 |
| Porcellidiidae | 0.10 | 0.12 | 4.89 | 1.28 | 13.21 | 59.33 |
| Santiidae | 0.07 | 0.07 | 3.96 | 1.40 | 10.70 | 70.03 |

**Raised vs. Surficial**: average dissimilarity = 72.94

| Family | Av.abund 1 | Av.abund 2 | Av.diss | Diss/sd | Contrib% | Cum.% |
| --- | --- | --- | --- | --- | --- | --- |
| Harpacticoida | 0.06 | 1.31 | 51.40 | 1.80 | 70.47 | 70.47 |

**Open vs. Surficial**: average dissimilarity = 65.50

| Family | Av.abund 1 | Av.abund 2 | Av.diss | Diss/sd | Contrib% | Cum.% |
| --- | --- | --- | --- | --- | --- | --- |
| Harpacticoida | 0.09 | 1.31 | 46.69 | 1.75 | 71.29 | 71.29 |

**Interstitial vs. Surficial**: average dissimilarity = 59.64

| Family | Av.abund 1 | Av.abund 2 | Av.diss | Diss/sd | Contrib% | Cum.% |
| --- | --- | --- | --- | --- | --- | --- |
| Harpacticoida | 0.17 | 1.31 | 42.29 | 1.73 | 70.91 | 70.91 |

**Raised vs. Emergent**: average dissimilarity = 82.65

| Family | Av.abund 1 | Av.abund 2 | Av.diss | Diss/sd | Contrib% | Cum.% |
| --- | --- | --- | --- | --- | --- | --- |
| Porcellidiidae | 0.08 | 0.01 | 17.38 | 2.64 | 21.03 | 21.03 |
| Amphipoda | 0.06 | 0.01 | 12.76 | 2.69 | 15.43 | 36.46 |
| Harpacticoida | 0.06 | 0.01 | 10.46 | 2.07 | 12.66 | 49.12 |
| Calanoida | 0.00 | 0.04 | 10.13 | 2.17 | 12.26 | 61.38 |
| Appendicularia | 0.00 | 0.02 | 5.81 | 2.14 | 7.02 | 68.41 |
| Pyramidellidae | 0.03 | 0.00 | 5.52 | 1.02 | 6.68 | 75.08 |

**Open vs. Emergent**: average dissimilarity = 86.31

| Family | Av.abund 1 | Av.abund 2 | Av.diss | Diss/sd | Contrib% | Cum.% |
| --- | --- | --- | --- | --- | --- | --- |
| Pyramidellidae | 0.11 | 0.00 | 16.57 | 1.87 | 19.20 | 19.20 |
| Porcellidiidae | 0.10 | 0.01 | 15.22 | 2.84 | 17.63 | 36.83 |
| Harpacticoida | 0.09 | 0.01 | 12.53 | 1.27 | 14.52 | 51.35 |
| Santiidae | 0.07 | 0.00 | 11.96 | 2.70 | 13.86 | 65.21 |
| Calanoida | 0.00 | 0.04 | 6.72 | 2.00 | 7.79 | 73.00 |

**Interstitial vs. Emergent**: average dissimilarity = 88.06

| Family | Av.abund 1 | Av.abund 2 | Av.diss | Diss/sd | Contrib% | Cum.% |
| --- | --- | --- | --- | --- | --- | --- |
| Harpacticoida | 0.17 | 0.01 | 19.63 | 2.57 | 22.29 | 22.29 |
| Pyramidellidae | 0.13 | 0.00 | 16.47 | 2.29 | 18.71 | 40.99 |
| Porcellidiidae | 0.12 | 0.01 | 15.20 | 3.74 | 17.26 | 58.26 |
| Santiidae | 0.07 | 0.00 | 9.34 | 1.63 | 10.60 | 68.86 |
| Calanoida | 0.00 | 0.04 | 5.36 | 2.03 | 6.09 | 74.95 |

**Surficial vs. Emergent**: average dissimilarity = 93.96

| Family | Av.abund 1 | Av.abund 2 | Av.diss | Diss/sd | Contrib% | Cum.% |
| --- | --- | --- | --- | --- | --- | --- |
| Harpacticoida | 1.31 | 0.01 | 58.08 | 2.11 | 61.82 | 61.82 |
| Porcellidiidae | 0.15 | 0.01 | 9.02 | 1.53 | 9.60 | 71.42 |

**Biomass**

**Raised**: average similarity = 55.53

| Family | Av.Abund | Av.Sim | Sim/SD | Contrib% | Cum.% |
| --- | --- | --- | --- | --- | --- |
| Porcellidiidae | 0.60 | 14.57 | 2.75 | 26.23 | 26.23 |
| Amphipoda | 0.51 | 13.72 | 3.27 | 24.71 | 50.95 |
| Harpacticoida | 0.47 | 12.61 | 4.30 | 22.70 | 73.65 |

**Open**: average similarity = 46.11

| Family | Av.Abund | Av.Sim | Sim/SD | Contrib% | Cum.% |
| --- | --- | --- | --- | --- | --- |
| Porcellidiidae | 0.70 | 8.56 | 3.81 | 18.56 | 18.56 |
| Santiidae | 0.73 | 8.35 | 2.97 | 18.10 | 36.67 |
| Pyramidellidae | 0.80 | 7.59 | 1.99 | 16.46 | 53.13 |
| Harpacticoida | 0.63 | 6.38 | 1.80 | 13.83 | 66.96 |
| Gobiidae | 0.66 | 4.04 | 0.67 | 8.77 | 75.73 |

**Interstitial**: average similarity = 52.37

| Family | Av.abund | Av.sim | Sim/sd | Contrib% | Cum.% |
| --- | --- | --- | --- | --- | --- |
| Harpacticoida | 1.01 | 10.74 | 2.43 | 20.51 | 20.51 |
| Pyramidellidae | 0.93 | 9.08 | 2.97 | 17.33 | 37.84 |
| Porcellidiidae | 0.84 | 9.01 | 3.71 | 17.21 | 55.05 |
| Santiidae | 0.69 | 6.87 | 1.24 | 13.12 | 68.17 |
| Amphipoda | 0.32 | 3.06 | 1.37 | 5.85 | 74.03 |

**Surficial**: average similarity = 51.11

| Family | Av.abund | Av.sim | Sim/sd | Contrib% | Cum.% |
| --- | --- | --- | --- | --- | --- |
| Harpacticoida | 3.05 | 22.29 | 1.81 | 43.62 | 43.62 |
| Porcellidiidae | 0.93 | 8.09 | 2.15 | 15.84 | 59.46 |
| Pyramidellidae | 0.78 | 6.67 | 2.18 | 13.04 | 72.50 |

**Emergent**: average similarity = 62.59

| Family | Av.abund | Av.sim | Sim/sd | Contrib% | Cum.% |
| --- | --- | --- | --- | --- | --- |
| Calanoida | 0.36 | 22.26 | 4.99 | 35.57 | 35.57 |
| Appendicularia | 0.23 | 14.47 | 2.50 | 23.12 | 58.70 |
| Harpacticoida | 0.14 | 6.86 | 1.31 | 10.96 | 69.65 |
| Ostracoda | 0.08 | 4.39 | 1.51 | 7.01 | 76.67 |

**Raised vs. Open**: average dissimilarity = 60.60

| Family | Av.abund 1 | Av.abund 2 | Av.diss | Diss/sd | Contrib% | Cum.% |
| --- | --- | --- | --- | --- | --- | --- |
| Santiidae | 0.05 | 0.73 | 6.82 | 2.92 | 11.26 | 11.26 |
| Gobiidae | 0.23 | 0.66 | 6.28 | 1.23 | 10.36 | 21.61 |
| Ophidiasteridae | 0.00 | 0.63 | 6.20 | 0.48 | 10.23 | 31.84 |
| Pyramidellidae | 0.24 | 0.80 | 6.03 | 1.52 | 9.95 | 41.79 |
| Galatheidae | 0.25 | 0.46 | 5.60 | 0.75 | 9.25 | 51.04 |
| Harpacticoida | 0.47 | 0.63 | 3.22 | 1.24 | 5.31 | 56.35 |
| Palaemonidae | 0.00 | 0.35 | 3.02 | 0.72 | 4.98 | 61.32 |
| Porcellidiidae | 0.60 | 0.70 | 2.74 | 1.23 | 4.52 | 65.85 |
| Amphiuridae | 0.04 | 0.36 | 2.67 | 0.39 | 4.40 | 70.25 |

**Raised vs. Interstitial**: average dissimilarity = 59.98

| Family | Av.abund 1 | Av.abund 2 | Av.diss | Diss/sd | Contrib% | Cum.% |
| --- | --- | --- | --- | --- | --- | --- |
| Pyramidellidae | 0.24 | 0.93 | 6.57 | 1.69 | 10.95 | 10.95 |
| Santiidae | 0.05 | 0.69 | 6.45 | 1.59 | 10.75 | 21.70 |
| Harpacticoida | 0.47 | 1.01 | 5.54 | 1.83 | 9.24 | 30.93 |
| Palaemonidae | 0.00 | 0.54 | 5.09 | 0.76 | 8.48 | 39.42 |
| Galatheidae | 0.25 | 0.38 | 4.96 | 0.67 | 8.26 | 47.68 |
| Gobiidae | 0.23 | 0.58 | 4.70 | 1.19 | 7.83 | 55.51 |
| Porcellidiidae | 0.60 | 0.84 | 3.12 | 1.23 | 5.21 | 60.71 |
| Amphiuridae | 0.04 | 0.39 | 2.77 | 0.38 | 4.61 | 65.33 |
| Nereididae | 0.03 | 0.26 | 2.26 | 1.03 | 3.76 | 69.09 |
| Amphipoda | 0.51 | 0.32 | 2.14 | 1.51 | 3.57 | 72.66 |

**Open vs. Interstitial**: average dissimilarity = 50.80

| Family | Av.abund 1 | Av.abund 2 | Av.diss | Diss/sd | Contrib% | Cum.% |
| --- | --- | --- | --- | --- | --- | --- |
| Gobiidae | 0.66 | 0.58 | 4.77 | 1.24 | 9.39 | 9.39 |
| Galatheidae | 0.46 | 0.38 | 4.65 | 0.72 | 9.16 | 18.55 |
| Palaemonidae | 0.35 | 0.54 | 4.41 | 0.98 | 8.67 | 27.22 |
| Ophidiasteridae | 0.63 | 0.00 | 4.27 | 0.48 | 8.41 | 35.63 |
| Harpacticoida | 0.63 | 1.01 | 3.58 | 1.42 | 7.04 | 42.67 |
| Amphiuridae | 0.36 | 0.39 | 3.51 | 0.47 | 6.91 | 49.58 |
| Pyramidellidae | 0.80 | 0.93 | 3.34 | 1.32 | 6.57 | 56.15 |
| Santiidae | 0.73 | 0.69 | 2.47 | 1.39 | 4.87 | 61.02 |
| Porcellidiidae | 0.70 | 0.84 | 1.99 | 1.22 | 3.91 | 64.93 |
| Nereididae | 0.04 | 0.26 | 1.65 | 0.98 | 3.26 | 68.19 |
| Aplysiidae | 0.00 | 0.29 | 1.48 | 0.33 | 2.91 | 71.10 |

**Raised vs. Surficial**: average dissimilarity = 64.93

| Family | Av.abund 1 | Av.abund 2 | Av.diss | Diss/sd | Contrib% | Cum.% |
| --- | --- | --- | --- | --- | --- | --- |
| Harpacticoida | 0.47 | 3.05 | 19.65 | 1.91 | 30.26 | 30.26 |
| Pyramidellidae | 0.24 | 0.78 | 5.09 | 1.56 | 7.84 | 38.10 |
| Porcellidiidae | 0.60 | 0.93 | 3.93 | 1.39 | 6.05 | 44.14 |
| Santiidae | 0.05 | 0.42 | 3.47 | 1.07 | 5.35 | 49.49 |
| Galatheidae | 0.25 | 0.17 | 2.80 | 0.70 | 4.31 | 53.79 |
| Palaemonidae | 0.00 | 0.27 | 2.38 | 0.88 | 3.67 | 57.46 |
| Gobiidae | 0.23 | 0.12 | 2.32 | 0.98 | 3.58 | 61.04 |
| Phyllidiidae | 0.00 | 0.30 | 2.24 | 0.33 | 3.45 | 64.49 |
| Fasciolariidae | 0.00 | 0.30 | 2.17 | 0.70 | 3.34 | 67.83 |
| Amphipoda | 0.51 | 0.44 | 2.03 | 1.21 | 3.13 | 70.96 |

**Open vs. Surficial**: average dissimilarity = 60.61

| Family | Av.abund 1 | Av.abund 2 | Av.diss | Diss/sd | Contrib% | Cum.% |
| --- | --- | --- | --- | --- | --- | --- |
| Harpacticoida | 0.63 | 3.05 | 14.77 | 1.79 | 24.37 | 24.37 |
| Gobiidae | 0.66 | 0.12 | 4.16 | 1.09 | 6.86 | 31.22 |
| Ophidiasteridae | 0.63 | 0.00 | 4.02 | 0.48 | 6.63 | 37.85 |
| Galatheidae | 0.46 | 0.17 | 3.29 | 0.67 | 5.43 | 43.29 |
| Pyramidellidae | 0.80 | 0.78 | 2.89 | 1.30 | 4.77 | 48.05 |
| Santiidae | 0.73 | 0.42 | 2.77 | 1.58 | 4.57 | 52.63 |
| Palaemonidae | 0.35 | 0.27 | 2.68 | 1.13 | 4.42 | 57.05 |
| Porcellidiidae | 0.70 | 0.93 | 2.61 | 1.36 | 4.30 | 61.35 |
| Phyllidiidae | 0.00 | 0.30 | 1.77 | 0.33 | 2.92 | 64.27 |
| Fasciolariidae | 0.01 | 0.30 | 1.75 | 0.72 | 2.88 | 67.15 |
| Amphiuridae | 0.36 | 0.00 | 1.71 | 0.33 | 2.83 | 69.98 |
| Amphipoda | 0.30 | 0.44 | 1.60 | 1.00 | 2.65 | 72.62 |

**Interstitial vs. Surficial**: average dissimilarity = 54.95

| Family | Av.abund 1 | Av.abund 2 | Av.diss | Diss/sd | Contrib% | Cum.% |
| --- | --- | --- | --- | --- | --- | --- |
| Harpacticoida | 1.01 | 3.05 | 12.40 | 1.79 | 22.57 | 22.57 |
| Palaemonidae | 0.54 | 0.27 | 3.70 | 1.03 | 6.73 | 29.30 |
| Gobiidae | 0.58 | 0.12 | 3.24 | 1.04 | 5.89 | 35.19 |
| Galatheidae | 0.38 | 0.17 | 2.88 | 0.57 | 5.24 | 40.43 |
| Santiidae | 0.69 | 0.42 | 2.85 | 1.37 | 5.18 | 45.62 |
| Pyramidellidae | 0.93 | 0.78 | 2.61 | 1.31 | 4.75 | 50.37 |
| Porcellidiidae | 0.84 | 0.93 | 2.30 | 1.34 | 4.19 | 54.56 |
| Amphiuridae | 0.39 | 0.00 | 1.82 | 0.33 | 3.31 | 57.88 |
| Phyllidiidae | 0.00 | 0.30 | 1.68 | 0.33 | 3.05 | 60.93 |
| Fasciolariidae | 0.00 | 0.30 | 1.64 | 0.70 | 2.98 | 63.91 |
| Nereididae | 0.26 | 0.10 | 1.54 | 1.07 | 2.81 | 66.72 |
| Amphipoda | 0.32 | 0.44 | 1.50 | 1.06 | 2.74 | 69.46 |
| Aplysiidae | 0.29 | 0.00 | 1.34 | 0.33 | 2.45 | 71.90 |

**Raised vs. Emergent**: average dissimilarity = 84.20

| Family | Av.abund 1 | Av.abund 2 | Av.diss | Diss/sd | Contrib% | Cum.% |
| --- | --- | --- | --- | --- | --- | --- |
| Porcellidiidae | 0.60 | 0.05 | 12.24 | 2.67 | 14.54 | 14.54 |
| Amphipoda | 0.51 | 0.07 | 10.19 | 3.03 | 12.11 | 26.65 |
| Calanoida | 0.01 | 0.36 | 8.22 | 2.66 | 9.76 | 36.41 |
| Harpacticoida | 0.47 | 0.14 | 7.74 | 2.15 | 9.19 | 45.60 |
| Gobiidae | 0.23 | 0.00 | 5.62 | 0.93 | 6.67 | 52.27 |
| Appendicularia | 0.00 | 0.23 | 5.51 | 2.55 | 6.54 | 58.81 |
| Pyramidellidae | 0.24 | 0.00 | 5.25 | 1.03 | 6.24 | 65.05 |
| Galatheidae | 0.25 | 0.00 | 5.08 | 0.57 | 6.04 | 71.08 |

**Open vs. Emergent**: average dissimilarity = 89.83

| Family | Av.abund 1 | Av.abund 2 | Av.diss | Diss/sd | Contrib% | Cum.% |
| --- | --- | --- | --- | --- | --- | --- |
| Pyramidellidae | 0.80 | 0.00 | 9.60 | 2.08 | 10.68 | 10.68 |
| Santiidae | 0.73 | 0.00 | 9.07 | 3.17 | 10.10 | 20.78 |
| Porcellidiidae | 0.70 | 0.05 | 8.28 | 2.67 | 9.22 | 30.00 |
| Gobiidae | 0.66 | 0.00 | 8.23 | 1.07 | 9.16 | 39.16 |
| Ophidiasteridae | 0.63 | 0.00 | 7.52 | 0.48 | 8.37 | 47.53 |
| Harpacticoida | 0.63 | 0.14 | 6.41 | 1.40 | 7.14 | 54.67 |
| Galatheidae | 0.46 | 0.00 | 5.16 | 0.55 | 5.74 | 60.41 |
| Calanoida | 0.02 | 0.36 | 4.37 | 2.20 | 4.87 | 65.28 |
| Janiridae | 0.31 | 0.00 | 3.95 | 1.56 | 4.40 | 69.67 |
| Palaemonidae | 0.35 | 0.00 | 3.60 | 0.72 | 4.01 | 73.68 |

**Interstitial vs. Emergent**: average dissimilarity = 90.38

| Family | Av.abund 1 | Av.abund 2 | Av.diss | Diss/sd | Contrib% | Cum.% |
| --- | --- | --- | --- | --- | --- | --- |
| Pyramidellidae | 0.93 | 0.00 | 10.38 | 2.71 | 11.49 | 11.49 |
| Harpacticoida | 1.01 | 0.14 | 10.13 | 2.25 | 11.21 | 22.69 |
| Porcellidiidae | 0.84 | 0.05 | 8.99 | 2.94 | 9.94 | 32.64 |
| Santiidae | 0.69 | 0.00 | 8.48 | 1.63 | 9.39 | 42.02 |
| Palaemonidae | 0.54 | 0.00 | 6.10 | 0.77 | 6.75 | 48.78 |
| Gobiidae | 0.58 | 0.00 | 5.58 | 1.01 | 6.17 | 54.95 |
| Galatheidae | 0.38 | 0.00 | 4.27 | 0.47 | 4.73 | 59.68 |
| Calanoida | 0.02 | 0.36 | 3.95 | 2.16 | 4.37 | 64.04 |
| Amphipoda | 0.32 | 0.07 | 3.22 | 1.49 | 3.56 | 67.60 |
| Amphiuridae | 0.39 | 0.00 | 2.79 | 0.34 | 3.09 | 70.69 |

**Surficial vs. Emergent**: average dissimilarity = 91.69

| Family | Av.abund 1 | Av.abund 2 | Av.diss | Diss/sd | Contrib% | Cum.% |
| --- | --- | --- | --- | --- | --- | --- |
| Harpacticoida | 3.05 | 0.14 | 26.20 | 2.50 | 28.58 | 28.58 |
| Porcellidiidae | 0.93 | 0.05 | 9.01 | 2.13 | 9.83 | 38.41 |
| Pyramidellidae | 0.78 | 0.00 | 8.12 | 2.15 | 8.86 | 47.27 |
| Santiidae | 0.42 | 0.00 | 4.66 | 1.10 | 5.08 | 52.35 |
| Amphipoda | 0.44 | 0.07 | 4.43 | 1.20 | 4.83 | 57.18 |
| Calanoida | 0.02 | 0.36 | 3.60 | 2.01 | 3.92 | 61.10 |
| Palaemonidae | 0.27 | 0.00 | 2.85 | 0.85 | 3.10 | 64.21 |
| Phyllidiidae | 0.30 | 0.00 | 2.57 | 0.33 | 2.80 | 67.01 |
| Appendicularia | 0.00 | 0.23 | 2.49 | 2.04 | 2.71 | 69.72 |
| Fasciolariidae | 0.30 | 0.00 | 2.48 | 0.71 | 2.70 | 72.42 |
